# Modelling the Time Evolution of Active Caspase-3 Protein in the Rat Lens after *In Vivo* Exposure to Ultraviolet Radiation-B

**DOI:** 10.1371/journal.pone.0106926

**Published:** 2014-09-22

**Authors:** Nooshin Talebizadeh, Zhaohua Yu, Martin Kronschläger, Per Söderberg

**Affiliations:** Gullstrand lab of Ophthalmology, Department of Neuroscience, Uppsala University, Uppsala, Sweden; University of Tennessee, United States of America

## Abstract

**Purpose:**

To introduce a model for the time evolution of active caspase-3 protein expression in albino rat lens up to 24 hours after in vivo exposure to low dose UVR in the 300 nm wavelength region (UVR-300 nm).

**Methods:**

Forty Sprague-Dawley rats were unilaterally exposed in vivo to 1 kJ/m^2^ UVR-300 nm for 15 minutes. At 0.5, 8, 16, and 24 hours after the UVR exposure, the exposed and contralateral not-exposed lenses were removed and processed for immunohistochemistry. The differences in the probability of active caspase-3 expression at four different time points after exposure were used to determine the time evolution of active caspase-3 expression. A logistic model was introduced for the expression of active caspase-3. The parameters for the exposed and the not exposed lenses were estimated for the observation time points.

**Results:**

The exposure to UVR-300 nm impacted on the parameters of the logistic model. Further, the parameters of the model varied with time after exposure to UVR-300 nm.

**Conclusion:**

The logistic model predicts the impact of exposure to UVR-300 nm on the spatial distribution of probability of active caspase-3 protein expression, depending on time.

## Introduction

Exposure to ultraviolet radiation is considered as a key risk factor for cataract development [1], [2]. Experimental exposure of the crystalline lens to ultraviolet radiation in the 300 nm wavelength region (UVR-300 nm) damages the lens epithelial cells [3]–[5]. The effects of ultraviolet radiation depend on the radiation dose, duration and wavelength [6]–[8]. Higher doses than the maximum tolerance dose (MTD_2.316_ = 3.65 kJ/m^2^) result in considerable opacities and induce cataract [9]. Lower doses lead to lower degrees of opacities in the lens and is more characterised by changes in lens proteins expression [10]. The changes in proteins expression regulate apoptosis and recovery of the lens damage [11]. Caspase-3 is one of the important proteins in the execution and completion of apoptosis [12], [13]. In the rat lens, exposure to UVR-300 nm damages the lens epithelial cells, induces apoptosis [3], [5], [14] and increases the expression of active caspase-3 protein in the lens epithelium [3], [10], [11], [15].

The spatial distribution of expression of active caspase-3 in lens epithelium in UVR-300 nm exposed lenses and not exposed lenses, was reported recently [10]. The purpose of the present study was to develop a mathematical model that describes the time dependent alteration of active caspase-3 expression as a consequence of in vivo exposure to low dose UVR-300 nm, as a function of cell position in the lens epithelium.

## Materials and Methods

**Figure 1:**
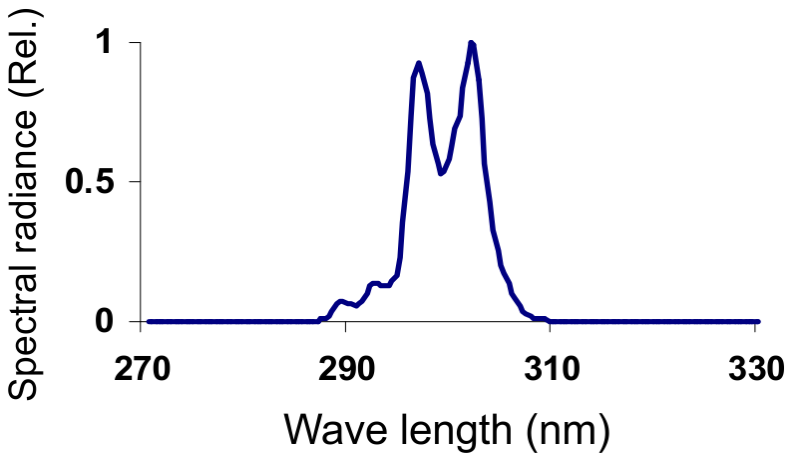
Relative spectral irradiance of ultraviolet radiation on the cornea.

This study is based on data collected in our previous study [10]. In the previous study, the expression of active caspase-3 was measured in the single layer of epithelial cells in 80 lenses (from 40 rats) at 0.5, 8, 16, or 24 hours after in vivo exposure to a total dose of 1 kJ/m^2^ (1.11 W, 15 minutes) UVR-300 nm. One eye from each animal was exposed to ultraviolet radiation. UVR in the 300 nm wavelength region was generated with a high pressure mercury arc lamp. The emerging radiation was collimated and then passed through a water filter and a double monochromator. The output radiation (Figure 1) centred around 300 nm (Full width half maximum  = 10 nm), was finally projected in a narrow beam covering the cornea of the exposed eye. The corneal irradiance during the exposure was measured before and after exposure with a thermopile calibrated to NIST (National Institute of Standards and Technology, USA) traceable source. The contralateral eye was covered.

Three mid sagital sections from each eye were observed under the microscope to measure the active caspase-3 expression in the lens epithelium. For each section, the expression of active caspase-3 was measured three times. The animals were kept and treated according to the ARVO Statement for the use of animals in ophthalmic and vision research. Ethical permission was obtained from the Uppsala University animal experiment ethics board (C 29/10).

In order to determine the distribution of active caspase-3 expression, the numbers of epithelial cells in each section were split into halves, assuming symmetry around the anterior pole of the lens. Each half was split into 15 segments. The average number of cells in each half of the lens was estimated to 213 cells. Each half is presented from number zero indicative of the anterior pole of the lens and number 213 indicative of the equator of the lens.

### Statistical parameters

The significance level and the confidence coefficient were set to 0.05 and 0.95, respectively.

## Results

### The model

**Figure 2:**
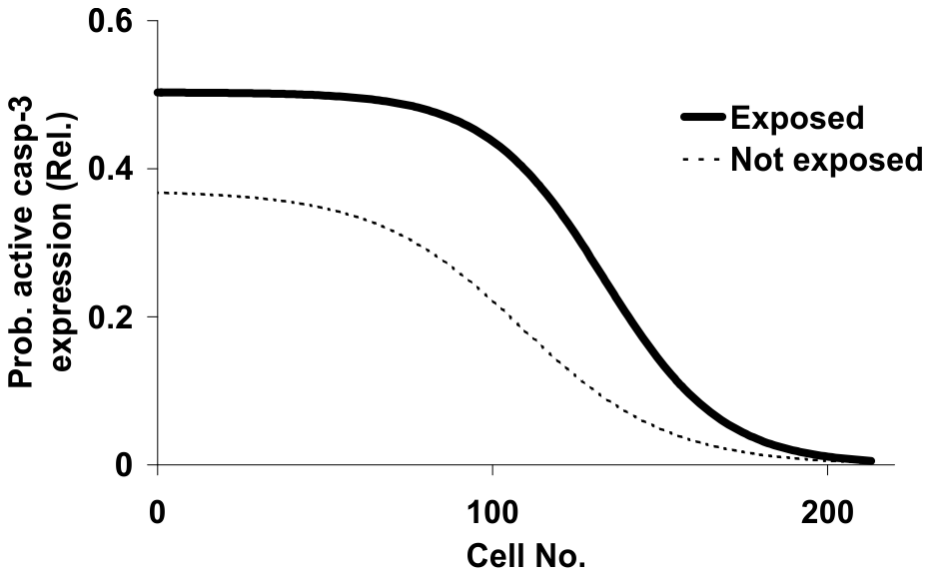
Model for the average probability of active caspase-3 expression in the lenses exposed to 1 kJ/m2 UVR-300 and contralateral not exposed lenses.

There was no expression of active caspase-3 at the equator in either of the exposed or the not exposed lenses. Active caspase-3 expression increased towards the anterior pole of the lens as a function of position in the lens epithelium. But the increase rate, as a function of position, was different in exposed lenses compared to contralateral not exposed lenses (Figure 2).

The probability of expression of active caspase-3 increased exponentially from the equator through an inflection point and then decreased towards an asymptote at the anterior pole of the lens. It is thus important to include a parameter that accounts for maximum expression of active caspase-3 at the anterior pole, a parameter for the rate of increase and a parameter for the inflection point of the rate in the model.

On the assumption that the increase of rate of active caspase-3 expression close to the equator, and the decrease of rate of active caspase-3 expression close to the anterior pole are the same, a simplified generalized logistic model was introduced. The model implies that the probability of active caspase-3 expression, *p(n)*, depends on n (segment number) and the parameters; the anterior pole asymptote probability of active caspase-3 expression, *ρ* (probability); the rate constant, *κ* (segment number.^−1^); the phase of the probability of active caspase-3 expression *η* (segment number), and a unit less proportionality parameter for the exponential factor, *φ*. (Eqn.1).
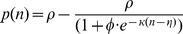
(1)


The inflection point can be calculated from the parameters estimated as indicated in Eqn.2.
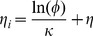
(2)


Probability of expression of active caspase-3 was fit as a function of segment number and is presented in figures with segment number transformed to cell number.

### Effect of exposure to UVR-300 nm

The probability of active caspase-3 expression as a function of cell position depends on exposure to UVR-300 nm. In the observed UVR-300 nm exposed lenses, the rate of expression of active caspase-3 increased more peripherally than in the contralateral not exposed lenses. Further, the asymptote of active caspase-3 was higher in exposed lenses than in the contralateral not exposed lenses (Figure 2).

**Figure 3:**
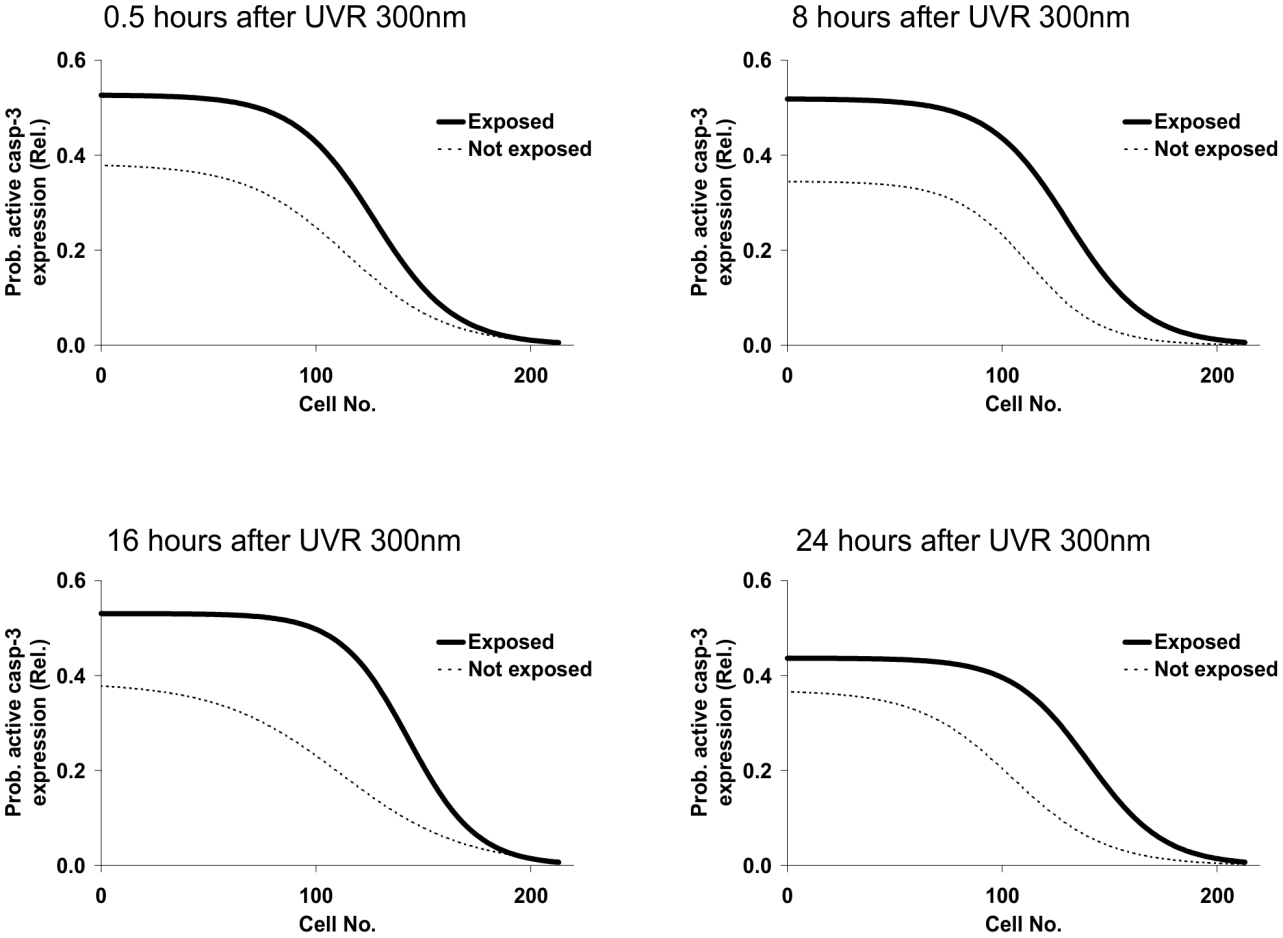
Probability of active caspase-3 expression in the exposed and the contralateral not exposed lenses as a function of cell number at 0.5, 8, 16 and 24 hours after exposure to 1 kJ/m^2^ UVR-300.

The expression of active caspase-3 as a function of cell number also varied as a function of time after UVR-300 nm exposure in the time window 0.5–24 hours (Figure 3).

### Time evolution as a function of cell position

**Figure 4:**
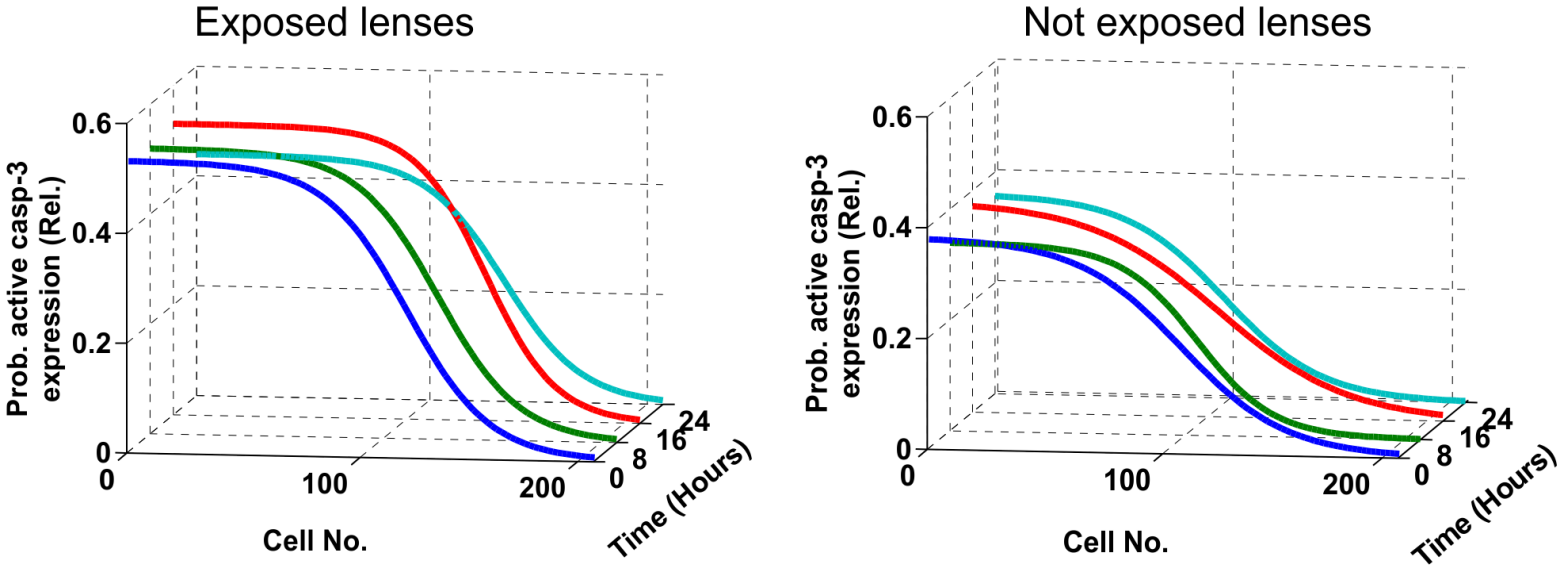
Probability of active caspase-3 expression after in vivo exposure to 1 kJ/m^2^ UVR-300 nm in the exposed and not exposed lenses as a function of cell number and time after exposure.

The expression of active caspase-3 as a function of cell position and time after exposure in exposed and contralateral not exposed lenses is shown in Figure 4.

The parameters, *ρ, κ, η and φ* (Eqn.1) were estimated by fitting the probability of expression of active caspase-3 as a function of position in each lens. Then, the averages of the four parameters at 0.5, 8, 16 and 24 hours after the exposure, respectively, were estimated (Table 1).

**Table 1:** Model parameters estimated for the distribution of probability of active caspase-3 expression as a function of cell number at 0.5, 8, 16 and 24 hours after in vivo exposure to 1 kJ/m^2^ UVR-300 nm.

Parameter	Exposure	Time (Hour)			
		0.5	8	16	24
Rate constant (Segment number ^−1^)	Exposed lenses	−0.79±0.09	−0.77±0.09	−0.89±0.01	−0.80±0.09
	Not exposed lenses	−0.61±0.20	−0.83±0.26	−0.50±0.10	−0.65±0.22
The phase of the probability of expression (Segment number)	Exposed lenses	2.9±0.6	3.0±0.8	3.4±0.3	3.1±0.8
	Not exposed lenses	7.2±1.0	6.6±0.9	5.2±0.5	5.9±0.8
Proportionality parameter for the exponential factor (Unitless) ×10^−2^	Exposed lenses	0.99±0.50	0.81±0.40	0.27±0.10	0.44±0.20
	Not exposed lenses	59.2±25.5	34.1±24.3	26.9±10.4	37.4±22.4
Anterior pole asymptote expression (Rel)	Exposed lenses	0.53±0.03	0.52±0.03	0.53±0.03	0.44±0.04
	Not exposed lenses	0.38±0.05	0.34±0.06	0.39±0.03	0.37±0.04

Intervals are 95% confidence interval for the means for parameters.

Degree of freedom  = 39.

An analysis of variance of the estimates of anterior pole asymptote parameters, with a fixed effect model (Appendix 1), indicated a variation among post exposure times for exposed lenses (Test statistics  = 7.83; F_3; 36; 0.95_ = 2.87), but no variation among non exposed lenses (Test statistics  = 0.8; F_3; 36; 0.95_ = 2.87). The estimates of the anterior pole asymptote parameters for not exposed contralateral lenses, were not analyzed with statistical inference since the exposed and the contralateral lenses originates from the same animal. A further analysis of potential contrasts among the post exposure times with orthogonal comparison [16] suggested a difference when 0.5 hour was tested versus 24 hours (Table 2).

**Table 2:** Orthogonal comparison of anterior pole asymptote probability of active caspase-3 expression.

Post-exposure time contrast	Degrees of freedom	Exposed Lenses	
		Estimated mean square ×10^−3^ (%)^2^	Test statistics
0.5 hr versus 24 hrs	1	40.58	16.45*
8 hrs versus 16 hrs	1	0.68	0.28
0.5 and 24 hrs versus 8 and 16 hrs	1	18.40	7.46*
Measurment error	36	2.47	

*Significance.

Significance limit: 4.11 (F_1; 36; 0.95_).

In addition, the analysis indicated a difference when 0.5 and 24 hours was compared to 8 and 16 hours (Table 2). The estimates for the other parameters were not statistically analyzed since all the other parameter estimates originates from the same animals and therefore are dependent.

In order to establish the time dependence of each parameter, the estimates of each parameter, *Y(t)*, at each time point, *t* (Hours), was fitted to a third order polynomial model with regression, assuming a random error, ε (Eqn.3).
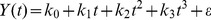
(3)


By introducing the dependence of the parameters on post exposure time into Eqn.1, and assuming rotational symmetry around the axis of the crystalline lens, a video of the time dependence of active caspase-3 evolution was generated (Video S1).

## Discussion

The current study intended to develop a mathematical model for the kinetics of active caspase-3 after exposure to UVR-300 nm.

In the present paper, the time evolution of active caspase-3 after exposure to UVR-300 nm was spatially resolved within the lens epithelium. We previously showed that exposure to a low dose of UVR-300 nm induces an increase in active caspase-3 expression detectable 0.5 hour after exposure. The expression of active caspase-3 continues to increase to a peak of expression at 16 hours, and then decreases at 24 hours [10]. There is one other study which reported the involvement of active caspase-3 in apoptosis induced by UVR-300 nm [14]. Galichanin et al showed the kinetics of caspase-3 mRNA expression in the rat lens after exposure to a double threshold dose of UVR-300 nm. The present study is the first study that shows the kinetic of active caspase-3 protein after exposure to a low dose of UVR-300 nm and spatially resolves the probability of active caspase-3 protein expression as a function of position in the epithelial array.

The current model (Eqn.1) was developed after a careful examination of scatter plots of probability of active caspase-3 expression as a function of position for each UVR exposed and not exposed lens. Both the exposed and the not exposed lenses showed a similar pattern of expression. The probability of expression increased from an apparent zero level asymptote at the equator through an inflection point in the mid region of the distance between the equator and the anterior pole. Then, the probability of expression increased with a declining rate towards an asymptote at the anterior pole of the lens. This pattern of expression led us to attempt to fit the spatial distribution of probability of expression of active caspase-3 to a logistic model (Figure 2). The data for both the exposed and the not exposed lenses fitted with a low residual error but with different estimates of the parameters (Table 1).

The model allows independent estimates of the parameters. The model implicates a very low or no expression at the equator and a saturation of expression at the anterior pole. Further the model implies that the rate of change is proportional to the position between the equator and the anterior pole and that the inflection point has a defined position in relation to the anterior pole and the equator. The finding that the absolute value of the rate constant tended to increase faster in the exposed lenses as compared to the not exposed lenses (Figure 2, Table 1) indicates that the contrast of active caspase-3 expression between the anterior pole and the equator increased due to the exposure.

Our observation that the phase of the distribution of active caspase-3 expression was shifted towards the anterior pole in the exposed lenses as compared to the not exposed lenses (Table 1) suggests that active caspase-3 expression became stronger towards the anterior pole in the exposed lenses. This is further emphasized, by the finding that the proportionality factor in the exposed lenses decreases with increasing post exposure time (Table 1). It is also reflected in the higher asymptote expression of active caspase-3 at the anterior pole in the exposed lenses as compared to the not exposed lenses (Table 1).

Our finding that the anterior pole asymptote probability for caspase-3 expression decreased towards 24 hours post exposure time is consistent with previous findings [10]. The previously reported initial increase of probability of active caspase-3 expression [10] was not reflected in the current study (Table 1, Table 2). The inconsistency in the findings is probably due to the fact that in the present study the probability of active caspase-3 expression at the anterior pole of the lens was estimated considering all the parameters that affect the asymptote. The fact that the anterior pole asymptote expression of active caspase-3 in the exposed lenses decreased at 24 hours after exposure (Table 1) suggests that the UVR-300 nm induced increase of active caspase-3 expression start to decline.

Active caspase-3 is known to be a key protein in apoptosis [3], [10], [11]. Thus, measurement of caspase-3 activation in lens epithelial cells is probably a good indicator of cellular damage in the lens epithelium. The currently developed model allows characterization of the spatial distribution of active caspase-3 in the lens epithelium. Determination of the time dependence of the parameters in the model provides spatial kinetic information on active caspase-3. Therefore, the model can be used to study the impact of experimental influence on the spatial kinetic information of active caspase-3. It is anticipated that the model will be useful both to understand the molecular biology of apoptosis and as a tool to study effects of damage to the lens epithelium such as oxidative insult.

Considering the strong epidemiological evidence that exposure to UVR is an important risk factor for cataract [1], [2], a better understanding of the molecular biology in lens epithelium exposed to UVR is important. We believe that the model developed will be a powerful tool to improve the knowledge on the molecular biology in the lens epithelium after exposure to UVR.

## Appendix

### Appendix 1 Model for analysis of variance of contrasts among time points

An estimate of a parameter, x_ij_, is the sum of the population mean, a term for the fixed variation among post exposure times, α_i_, and a term for the random variation among animals, ε_j(i)_ (Eqn. A.1).
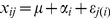
(A.1)


## Supporting Information

Video S1 **Time evolution of active caspase-3 expression after in vivo exposure to 1 kJ/m^2^ UVR-300 nm.**
(MOV)
